# Indigenous Cultural Safety Training for Applied Health, Social Work, and Education Professionals: A PRISMA Scoping Review

**DOI:** 10.3390/ijerph20065217

**Published:** 2023-03-22

**Authors:** Tammy L. MacLean, Jinfan Rose Qiang, Lynn Henderson, Andrea Bowra, Lisa Howard, Victoria Pringle, Tenzin Butsang, Emma Rice, Erica Di Ruggiero, Angela Mashford-Pringle

**Affiliations:** 1 Dalla Lana School of Public Health, University of Toronto, Toronto, ON M5T 3M7, Canada; 2 Department of Psychology, University of Toronto Mississauga, Mississauga, ON L5L 1C6, Canada; 3 Department of Clinical Studies, University of Guelph, Guelph, ON N1G 2W1, Canada; 4 Waakebiness Institute for Indigenous Health, Dalla Lana School of Public Health, University of Toronto, Toronto, ON M5T 3M7, Canada; 5 Centre for Global Health, Dalla Lana School of Public Health, University of Toronto, Toronto, ON M5T 3M7, Canada

**Keywords:** cultural safety, cultural competence, Indigenous health, Aboriginal health, health education, nursing, medicine, allied health, anti-racism, meta-synthesis

## Abstract

Anti-Indigenous racism is a widespread social problem in health and education systems in English-speaking colonized countries. Cultural safety training (CST) is often promoted as a key strategy to address this problem, yet little evidence exists on how CST is operationalized and evaluated in health and education systems. This scoping review sought to broadly synthesize the academic literature on how CST programs are developed, implemented, and evaluated in the applied health, social work and education fields in Canada, United States, Australia, and New Zealand. MEDLINE, EMBASE, CINAHL, ERIC, and ASSIA were searched for articles published between 1996 and 2020. The Joanna Briggs Institute’s three-step search strategy and PRISMA extension for scoping reviews were adopted, with 134 articles included. CST programs have grown significantly in the health, social work, and education fields in the last three decades, and they vary significantly in their objectives, modalities, timelines, and how they are evaluated. The involvement of Indigenous peoples in CST programs is common, but their roles are rarely specified. Indigenous groups must be intentionally and meaningfully engaged throughout the entire duration of research and practice. Cultural safety and various related concepts should be careful considered and applied for the relevant context.

## 1. Introduction

There is substantial evidence to suggest that anti-Indigenous racism is a widespread health and social problem in Canada. While the term, Indigenous, refers to a variety of Aboriginal groups around the world [1], in Canada, Indigenous refers to the country’s first inhabitants, namely, First Nations, Inuit, and Métis peoples, as established in Section 35 of the Canadian Constitution [2]. Racism may be understood as “racist ideologies, prejudiced attitudes, discriminatory behaviour, structural arrangements and institutionalized practices resulting in racial inequality as well as the fallacious notion that discriminatory relations between groups are morally and scientifically justifiable” [3]. According to several population-based studies undertaken with Indigenous peoples across Canada, between 39% and 43% of respondents reported experienced racism [4,5,6,7]. Systemic anti-Indigenous racism in health service organizations across the country is equally problematic. The investigations into the deaths of Brian Sinclair in Manitoba in 2008 [8] and Joyce Echaquan in Quebec in 2020 [9], coupled with the alarming findings from the anti-Indigenous racism investigation in British Columbia’s health care system in 2020 [10] collectively demonstrate the insidious nature of anti-Indigenous racism in Canada’s health systems. Not only is racism a serious challenge for Indigenous peoples while accessing care, but also it has profound negative impacts on their likelihood of accessing future care [10,11,12,13,14,15,16,17].

Beyond the health sector, research evidence also points to widespread anti-Indigenous racism in Canada’s social work [18,19] and Kindergarten to Grade 12 education systems [20,21]. Moreover, Indigenous peoples have been identified as the most disadvantaged with respect to accessing education in English speaking, colonized countries such as Canada, United States, Australia, and New Zealand [20].

In 2015, Canada’s Truth and Reconciliation Commission (TRC) recognized the legacy and impact of residential schools on Indigenous peoples across the country [22] and put forth 94 Calls to Action, including for the health, social work/child welfare, and education systems. These calls included providing cultural competency training for health professionals to address unconscious bias and systemic racism and developing culturally appropriate education curricula, while building student capacity for intercultural understanding, empathy, and mutual respect [23]. The calls also set out the need to ensure that social workers who conduct child-welfare investigations be properly educated and trained about the history and impacts of residential schools on children and their caregivers. In terms of education systems, the TRC recommended that resources be provided to ensure Indigenous schools utilize Indigenous knowledge and teaching methods in the classroom and that relevant teacher-training needs be identified to ensure such knowledge and methods are implemented.

Initiatives such as Canada’s TRC, along with similar federal government initiatives in Australia and New Zealand, have led to the emergence of new training programs to facilitate cultural safety and competence and, ultimately, to eliminate anti-Indigenous racism and discrimination in health, social work, and education systems [24,25]. However, given the nascency of the field of Indigenous cultural safety training combined with the need to expand the availability of such training to address anti-Indigenous racism, there is an urgent need to synthesize and understand existing evidence on how such training programs are conceptualized and developed, as well as how they are implemented in practice and evaluated for impact.

## 2. Objectives

Our team sought to synthesize the academic literature that conceptualizes and/or operationalizes Indigenous cultural safety training within the fields of health, social work, and education. The aim of this study was to answer the following three questions: (1) What is the general state of knowledge on Indigenous cultural safety training in the applied fields of health, social work, and education? (2) What methods are used to develop, implement, and evaluate Indigenous cultural safety training for students and professionals in the applied fields of health, social work, and education? (3) What content themes are included in existing Indigenous cultural safety training program for students and professionals in the fields of health, social work, and education?

## 3. Methods

### 3.1. Protocol and Registration

This study involved a scoping review that included the PRISMA extension protocol for scoping reviews (PRISMA-Scr) proposed by Tricco et al. [26]. A detailed research protocol for this scoping review is published in *Social Science Protocols* doi.org/10.7565/ssp.2020.2815 [27], an open-access online journal platform. The authors of this paper chose not to register this study, both because of the rigid methodological requirements for registration, which do not align well with Indigenous research approaches, and because no other Indigenous studies were registered at the time. Indigenous cultural safety is a nascent area of research and practice, and the goal of this review was to achieve a comprehensive search. Our analysis sought to draw out insights that could inform Indigenous cultural safety training for professionals in the applied fields of health, social work, and education, which work with Indigenous peoples in Canada.

### 3.2. Eligibility Criteria

We reviewed the global academic literature on Indigenous cultural safety training that included but was not limited to Indigenous peoples in Canada. Inclusion criteria: This review was restricted to articles about Indigenous cultural safety in health, social work and education in British colonial settler nation states, including Australia, New Zealand, Canada, and the United States. All peer-reviewed primary research articles on the topic of Indigenous cultural safety within the fields of health, social work, and education that were reported in the academic literature between 1996 and 2020 were included. The review dates were selected to ensure that the full history of the term “cultural safety”, first coined in 1996 [28], was captured. This review included all articles published in or translated into English. The exclusion criteria included articles published prior to 1996; articles published in countries other than New Zealand, Australia, United States, or Canada; articles focused on fields other than health, social work, or education; and research published as non-academic literature or in languages other than English.

### 3.3. Information Sources

To identify potentially relevant literature in health, social work, and education, the following five bibliographic databases were searched from 1996 to 2020: Medline, EMBASE, CINAHL, ERIC, and ASSIA. These databases were selected to capture the fields of health, education, and social work as aligned with the focus of this review. The search strategies were developed by an experienced librarian at the university where the authors are affiliated and further refined through team discussion.

### 3.4. Search Strategy

A three-step search strategy [29] was utilized for this review. The first step involved a limited search of two initial databases, Medline and EMBASE, followed by an analysis of subject headings and search terms based on identified titles and abstracts. A second search was then conducted using all identified subject headings and keywords across all five databases (see Table 1: Search Strategy).

### 3.5. Selection of Sources of Evidence

The selection of studies for this review was performed in Covidence independently by two reviewers and involved a four-stage process. The first stage of screening involved identifying and removing duplicate studies from Covidence. The second stage of screening involved reviewing the titles and abstracts of the remaining articles and applying the inclusion and exclusion criteria. Any disagreement between the two reviewers on applying the criteria was resolved through discussion.

### 3.6. Data Charting Process

A data-charting tool was developed for this study with input from several co-authors and a librarian at the university where the authors are affiliated. The data-charting tool was developed in Google Spreadsheet, and several decisions were made to establish the focus, nature, and scope of data to be extracted, including (1) to cut and paste entire “chunks” of text verbatim into the tool rather than paraphrasing the data; (2) to include article page numbers associated with data on cultural safety concepts and critiques; and (3) to include both the study aim, along with the research question if explicitly stated and relevant to the study. The data-charting tool was then piloted by all members of the review team based on two pre-selected articles. Several procedural decisions were made during the pilot stage to further articulate the scope of data to chart, both for existing data points and for two new data points added during this stage. These decisions included (1) whether the paper included Indigenous authors or Elders; (2) identifying whether a data collection or evaluation tool was included or referenced; (3) specifying that “training modality” referred to the format (e.g., in-person, online, etc.) and “training components” related to the topics covered; (4) including only “yes” or “no” on four specific data points; (5) specify that “duration of time the training program has been running” was in relation to the study publication year; (6) adding “recommendations outlined in the article”; and (7) adding “stated limitations of the evaluation”.

The data-charting process involved charting each article twice, and this was undertaken between August 2020 and June 2021. The first author independently completed one round of data-charting, and the second round was completed by a team of six reviewers independently. Several questions arose among the reviewers throughout the data charting process concerning the nature and amount of data to be extracted and charted, and these issues were generally resolved through discussion as a team. Any disagreements between two reviewers were resolved through dialogue or further adjudication by a third reviewer where necessary.

### 3.7. Data Items

The items included in the data-charting tool were developed deductively and addressed four main themes. The first theme included Article Characteristic for Indigenous cultural safety training for the sources of evidence included in the review, namely, bibliographic details; country of study; whether author(s) identified as Indigenous; study aim/objective(s); target field(s)/discipline(s) (i.e., medicine/physicians; nursing/midwifery; allied health; health, general; education; and/or social work); and target population (students and/or professionals). The second theme concerned Cultural Safety Concepts, Critiques and Rationale, which included cultural safety and related concepts either articulated or cited by study authors, along with critiques of the concepts, and the rationale given for why cultural safety is necessary in the fields of health, education, and social work.

The third theme involved the Characteristics of Cultural Safety Training described in the sources of evidence. Data themes included who sponsored and developed the training program, whether Indigenous scholars and practitioners or knowledge keepers were involved in the development process, and the roles of community partners who were engaged. Other themes included who delivered and received the training program, along with details of the training program itself—such as the objectives, delivery modality, component descriptions, delivery duration and timeline, details of any post-program support, and the programs’ stated limitations. The fourth and final theme addressed Evaluation Details of Cultural Safety Training. These data involved the evaluation objectives, data collection methods, type(s) of data collected (i.e., qual, quant, and both), evaluation results, stated evaluation limitations, recommendations for future cultural safety training programs, and whether a data collection tool was provided (see Appendix A).

### 3.8. Synthesis of Results

Once the data were charted, the data synthesis process was undertaken in three stages and carried out by three reviewers. The first stage involved analyzing both extractions of data for each theme to identify and resolve any discrepancies. All inconsistencies that were discovered were resolved through discussion and/or revisiting the sources of data where needed. The second stage involved analyzing data on the general characteristics of Indigenous cultural safety within the studies selected for the review, a process which involved categorizing the data within each data theme and then counting the frequency of findings within each category. The third stage involved synthesizing data that described a cultural safety training and/or the evaluation of a cultural safety training. The last stage involved synthesizing the data within each theme to create an overview of the data characteristics of Indigenous cultural safety, then categorizing the data within each theme into distinct groups and counting the frequency of findings within each category. Finally, the process of data synthesis also involved excluding or refocusing some of the data themes that were introduced in the protocol paper and proposed as part of this study. The reasons for these changes were varied and are set out in Section 4.4: Details of Indigenous Cultural Safety Training Intervention Evaluations.

## 4. Results

### 4.1. Selection of Sources of Evidence

**Figure 1:**
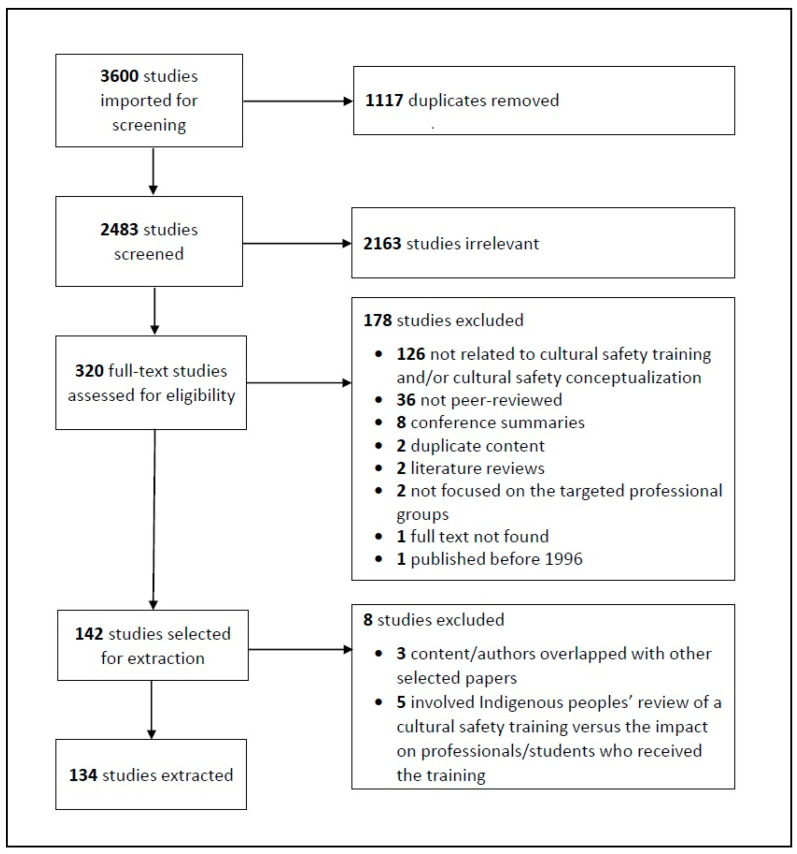
Prisma Flow Diagram.

The database searches were conducted in May 2020 and resulted in a total of 3600 sources, which were imported into Covidence online screening software (see Figure 1: Prisma Flow Diagram). Covidence detected and removed 1117 duplicate articles and 2153 irrelevant studies. In the end, 320 full-text articles were screened and assessed for eligibility within Covidence by two reviewers. Through this process, an additional 178 were excluded for the following reasons: (a) not related to cultural safety training and/or cultural safety conceptualization (*n* = 126); (b) not peer reviewed (*n* = 36); (c) conference summary (*n* = 8); (d) duplicate content (*n* = 2); (e) literature review (*n* = 2); (f) not focused on our target professional groups (*n* = 2); (g) full text of article not found/available (*n* = 1); and (h) published before 1996. While 142 remaining studies were selected for inclusion, an additional eight articles were removed during the process of data charting because either the authors and content of these articles overlapped significantly with other selected articles (*n* = 3), or the paper involved Indigenous peoples’ review of a cultural safety training but did not focus on the targeted professionals or students and their experiences with the training (*n* = 5). Data from a total of 134 included full-text articles, including quantitative and/or qualitative research studies as well as expert opinion pieces, were extracted for this study (for a complete list of studies selected, see Appendix B).

### 4.2. General Characteristics of Sources of Evidence

Table 2 presents the general characteristics of the 134 sources of evidence selected for this review. The citations for each finding presented in this table are included in Appendix C.

### 4.3. Details of Indigenous Cultural Safety Training Interventions

Of the papers that described an Indigenous cultural safety training program (*n* = 69), more than two-thirds mentioned the involvement of Indigenous peoples in the training development process (*n* = 48; 70%) (see citations for these finding in Appendix D). A wide variety of education approaches to delivering cultural safety content were illustrated as part of these interventions, with all having described more than one modality. Teaching/lectures was the most common training modality, described in almost a third of papers (*n* = 21; 30%). Workshops (*n* = 18; 26%), discussions sessions (*n* = 16; 23%), and immersive experiences/community visits (*n* = 13; 19%) were also common, detailed by an estimated quarter to approximately a fifth of relevant articles.

Following these modalities, storytelling/yarning reflective exercise/practice online content, and video/videocasts were each described by an eighth of chosen studies (*n* = 8; 12%). Also common among training modalities were clinical placements, case-studies, tutorials, and workbooks/readings, each of which were included as part of a tenth of interventions (*n* = 7; 10%). Finally, Indigenous Elders/mentors/community supports (*n* = 6; 4%), course-based modules (*n* = 5; 4%), and group work (*n* = 4; 3%) were each described by a few papers. The least common activities mentioned were drama/role-play exercises, and a visit to a gallery/museum, each mentioned by a couple of articles (*n* = 2; 3%). Notably, eight papers (12%) described the provision of follow up support for learners of cultural safety beyond the primary training period.

With respect to the training timeline, the largest proportion of articles detailing a cultural safety training intervention described the training as lasting 1–5 days/sessions (*n* = 15; 22%). Following this, in approximately one-sixth of selected articles, the training involved part of a one-semester undergraduate course (8–25 h) (*n* = 12; 17%). The shortest training involved a one-off lecture/workshop/cultural visit, which was mentioned in an estimated one-sixth of articles (*n* = 10; 15%), followed by a 1–5 week immersive experience in one of ten articles (*n* = 7; 10%). The longest training experiences involved embedded content across a four-year bachelor’s degree (unknown total hours) followed by training of 6–10 days/sessions, with each of these approaches occurring in one of ten articles (*n* = 6; 9%). Three training durations, namely, 3–12 months of immersion, a one-semester undergraduate course (42 h), and a partial undergraduate course over two semesters (50–52 h), each occurred in an estimated one-fifth of papers (*n* = 14; 20%). The two least frequent training timelines were included in one article each (*n* = 1; 1%), namely, an intensive course over two semesters (148 h), and two years of content embedded in a health service organization. Nearly one in five articles did not include details on the duration of cultural safety training (*n* = 13; 19%).

In terms of delivering cultural safety training interventions, university professors/administrators were mentioned in approximately twenty-five articles that described cultural safety training (25; 36%). Clinicians/clinical instructors (health professions) (*n* = 20; 30%), and Indigenous Elders/mentors/cultural educators/community/organizations (*n* = 19; 28%) were each mentioned in approximately one-third of relevant articles. University/college lecturers/educators delivered the training in an estimated one-fifth of articles (*n* = 15; 22%), followed by researchers in approximately one in twenty-five articles (*n* = 5; 7%). Allied health professionals and consultants were each mentioned in the context of training delivery in only a few articles (*n* = 3; 4%). Two articles provided no details on who delivered the training, while one article described non-clinician health staff as responsible for training delivery. Finally, half of the articles that described cultural safety training reported that Indigenous people(s) were involved in delivering the intervention (*n* = 35; 51%). Indigenous peoples involved in delivering cultural safety training includes both those categorized as “Indigenous Elder/Mentor/Cultural Educator/Community/Community organization” as well as those in other relevant categories listed immediately above.

### 4.4. Details of Indigenous Cultural Safety Training Intervention Evaluations

Nearly one-third of papers selected for this review described an evaluation of an Indigenous cultural safety training intervention (*n* = 61; 46%). The citations of these finding are included in Appendix E. These particular studies involved a broad range of objectives, with the largest proportion of papers (*n* = 19; 31%) aimed broadly at understanding leaners’ general experiences, perceptions, needs, and/or preferences related to a cultural safety training in which they participated. Another paper similarly focused on understanding perceptions of a given training intervention but from the perspective of Indigenous community members rather than participants (*n* = 1; 2%).

Other common evaluation approaches involved exploring participants’ reports of learning experiences, outcomes, and/or barriers as intervention outcomes (*n* = 16; 26%), and the new skills, behaviours, and/or practices that learners acquired (*n* = 15; 25%). Similarly common, reported in nearly one-fifth of relevant papers, involved understanding learners’ new knowledge, awareness, attitudes, and confidence since the intervention (*n* = 11; 18%). Other evaluation approaches included learners’ perceptions about Indigenous peoples and/or their health issues, as well as learners’ receptivity and/or resistance to intervention content, each mentioned in one intervention evaluation (*n* = 1; 2%). A less common approach to evaluating cultural safety training interventions among the selected articles involved exploring processes, and specifically intervention planning and implementation processes (*n* = 2; 3%), and leaners’ behaviour change processes (*n* = 2; 3%). Another paper looked at the relationship between learners’ intervention participation (intervention exposure) and their perceptions of Indigenous peoples (the outcome). Finally, several papers considered the context wherein the training intervention was implemented, specifically, the organizational context as an enabling factor (*n* = 5), the impact of the training on organizational processes and priorities (*n* = 3), and new organizational policies, positions, capacity, and/or mandate related to the indigenous cultural safety training (*n* = 2). In terms of the methodological approaches adopted to evaluate cultural safety training interventions, just over half of relevant articles described utilizing both qualitative and quantitative methodologies (*n* = 32; 52%). Another third of articles outlined the use of qualitative methods only (*n* = 20; 33%), while approximately one in six papers used quantitative methods only as part of their intervention evaluations (*n* = 9; 15%). A broad range of research methods was taken up to evaluate the influence and/or impact of cultural safety training interventions on participants. Data collection methods prior to the intervention involved pre-surveys (*n* = 13; 21%), only four of which included open-ended, qualitative questions (*n* = 6; 8%). Less common were pre-focus groups (*n* = 2; 3%) and a pre-test (*n* = 1; 2%). A small number of evaluation papers undertook observations/reflections half way through the interventions (*n* = 4; 6%).

The greatest range of data collection methods occurred immediately following the intervention, with post-surveys being the most common approach, mentioned by two-thirds of relevant articles. A large proportion of these post-surveys focused only on quantitative data (*n* = 24; 39%), while others also included open-ended qualitative questions (*n* = 12; 20%) Participant interviews were also common following an intervention, also mentioned in a fifth of relevant articles (*n* = 12; 20%). Oral or written learner feedback (*n* = 6; 10%), and post-focus groups (*n* = 6; 10%) were each mentioned in a tenth of papers describing a training evaluation, followed by learner reflections or case studies (*n* = 4; 6%) and learner journal entries or digital storytelling (*n* = 3; 5%), as described in a quarter of relevant papers. A post-test, talking circles, analysis of developed curriculum, and post-researcher observations/reflections were each mentioned in only one relevant study (*n* = 1; 2%). Finally, a small number of relevant studies involved data collection after a period of delay following a cultural safety training intervention. These methods included a delayed post-survey undertaken at various stages between 3 and 55 months following the intervention (*n* = 2; 3%), while a third paper described delayed learner reflections collected at 1–2 weeks following the intervention (*n* = 1; 2%). Finally, of the papers that described a cultural safety training intervention, only approximately half of these papers presented the limitations of the methods chosen for the intervention evaluations (*n* = 33; 54%).

The review team chose to exclude or refocus some of the data themes that were introduced in the protocol paper and proposed as part of this review study. The reasons for these changes were varied. First, data related to some of the data themes were largely homogenous. These themes included, for example, the rationale for providing training on cultural safety (or related) concepts—reasons which overwhelmingly involved reducing disparities between Indigenous people and relevant settler populations on health outcomes (for health-related papers) or education achievement (for education related papers). Data were also not presented in this paper on themes wherein the data were not described consistently across selected studies. This included themes such as description of training components, number of people who completed the training, and details of post-training supports provided. Similarly, data on who sponsored or developed the training intervention, as well as the duration of the training intervention and whether it was implemented in rural or urban settings, were largely incomplete, with a relatively small proportion of articles including fulsome descriptions for these themes. For its part, data on “the aim/objective” of the evaluation tended to be very general, including reasons such as “to evaluate a cultural safety training intervention”, which was therefore replaced with “evaluation focus”, which instead centred on the nature of data collected (i.e., participants perceptions, intervention processes or outcomes, etc.). Finally, the evaluation objectives and recommendation were not analyzed and included in this review because these data were rich in description, and synthesizing the findings would require undertaking content analysis, which the authors determined was beyond the scope of this review given the timeframe required to complete the study.

## 5. Discussion

### 5.1. Summary of Evidence

This study sought to synthesize the state of knowledge on Indigenous cultural safety training in the applied fields of health, social work, and education. This paper sets out the broad range of approaches used to develop, implement, and evaluate these training programs, along with the substantive themes included in the trainings.

This study found a significant growth in research about Indigenous cultural safety training in the three broad fields over the last few decades, and primarily within the last ten years. Half of the papers selected described cultural safety training, with the greatest share of these articles targeting the field of nursing/midwifery followed by medicine/physicians. This finding is unsurprising considering that nurses/midwives tend to comprise the largest share of the health service workforce, followed by physicians [30]. The education field, for its part, comprised only a tenth of selected articles, suggesting that much more is needed to improve access to cultural safety training for educators. Across the four broad fields, approximately half of the population targeted by the articles were students while the other half were professionals. This is an important finding as it suggests that not only are students learning about cultural safety within their post-secondary education programs, but so are instructors, mentors, and employers working in health, social work, and education fields. This may indicate that professionals have the capacity to contribute to enabling environments for new graduates to practice cultural safety when they begin their careers.

A substantial variety of training modalities was apparent in the training interventions, with teaching or lectures, workshops, and immersive experiences or community visits among the most common. Similarly, there was a huge range in the timeline or duration of cultural safety trainings. Interventions varied from several individual sessions to one or more months of immersive experience, to fully embedded content within a post-secondary course or throughout an entire undergraduate degree program. Even the actors who delivered the training ranged significantly, from university professors and health service clinicians, to lecturers, researchers, and consultants.

There was little consistency in the objectives and methods to evaluate Indigenous cultural safety training interventions. Some evaluations focused on the experiences of learners and how they made sense of those experiences, while others looked at specific outcomes or processes (or the relationships between these two factors). There were also evaluations that considered the contexts within which the interventions were implemented and how these contexts influenced the interventions. Methodological approaches chosen for intervention evaluations also varied widely, including qualitative, quantitative, and mixed methods approaches. Most studies collected data after a cultural safety intervention, while fewer considered relevant data prior to, during, or following a delay after an intervention. Pre- and post-surveys were the most common method adopted (with some including open-ended questions), followed by interviews, focus-groups, and methods involving written reflections, case studies, journal entries, and/or storytelling. Notably, only half of the studies included set out the limitations of the methods chosen for intervention evaluations.

Only a fifth of papers selected for this review included a named Indigenous author, while an estimated one-tenth of papers failed to specify even the broad Indigenous population relevant to the study (e.g., First Nations, as in Canada). Moreover, while a substantial proportion of these training interventions involved Indigenous people(s) in the development processes, the specific roles they undertook were rarely mentioned. Similarly, approximately half of the training interventions involved Indigenous Elders, experts, and other community members in the training delivery process, yet how they contributed to the training vis-à-vis other actors involved was not clearly articulated. These findings highlight the need to ensure Indigenous voices are central to identifying and prioritizing the content and approaches to be included in Indigenous cultural safety trainings [31]. Moreover, a greater commitment to meaningfully engage Indigenous communities on projects concerning them is urgently needed by researchers and educators working to advance cultural safety in the health, social work, and education fields [31,32].

Only half of the selected papers described and/or cited a cultural safety or similar concept. This is a somewhat surprising finding given that this review sought to understand the concept of cultural safety and how training interventions have attempted to facilitate its achievement. It is notable that many different concepts and definitions related to cultural safety emerged from the selected studies and were often used interchangeably. These concepts, in part, included cultural competence, cultural sensitivity, cultural responsiveness, cultural knowledge, cultural awareness, and cultural humility. Confusion related to these cultural concepts in selected studies was made worse by several factors, including (1) few attempts to justify why a given concept was chosen over another; (2) original sources for these concepts tended to not be clearly cited; and (3) little demonstrated understanding and contextualization of the literature wherein these concepts emerged. Similarly, and perhaps unsurprisingly given the current nascent state of this literature, less than a fifth of papers provided a critique of a cultural safety or similar concept.

There is evidence to suggest there is some convergence around the geopolitical origins of two concepts, namely, cultural safety and cultural competence. Cultural safety emerged in New Zealand by a Māori nurse working within the health system at a time when the country was primarily bi-racial, comprising Indigenous peoples and Western European settlers [28]. Cultural competence, for its part, is a term developed in the United States within the transcultural nursing movement and in response to nurses’ lack of understanding of the unique health needs of immigrants [33]. In addition to the convergence of these two concepts, Lauren Baba, a Canadian researcher at the National Collaborating Centre for Indigenous Health in British Columbia, published a 2013 report that draws upon multiple sources to conceptualize and articulate the distinctions between cultural safety, cultural competence, and other similar concepts, including cultural awareness and cultural sensitivity [34]. These four cultural related concepts and their definitions are outlined in Table 3 (Defining Cultural Safety, Cultural Competence, and Similar Concepts).

Both academic research involving these cultural related concepts and training programs that seek to facilitate their development would benefit from considering, comparing, and contrasting the concepts set out by Baba [34]. Not only would consolidating these terms across the literature work to further develop the scholarly work in this area, but also it would facilitate the advancement of the field more broadly by furthering the understanding of which concepts are preferable in different contexts and how to ensure their achievement through targeted training programs. This field of research would also benefit from advancing understanding on how these different concepts relate to one another in practice, how they should be applied, and the merits and drawbacks of each. Moreover, careful attention must be paid to the contexts in which these concepts are applied given the unique socio-political and historical situations in which some of them have emerged [28,33].

The increased attention to cultural safety and related concepts has been fueled by national initiatives to address the historical injustices against Indigenous peoples. For example, Canada’s Truth and Reconciliation Commission’s Calls to Action (2015) set out the need for cultural safety training for healthcare professionals, and federal funding for post-secondary institutions and educators to establish national research programs in collaboration with Indigenous peoples to advance Reconciliation [23]. A similar 2008 initiative in Australia, entitled Closing the Gap [24], called for multi-sectoral action in collaboration with Aboriginal communities to improve Indigenous health, which was followed in 2019 by the development of a National Indigenous Agency in 2019 responsible for leading and coordinating the implementation of the strategy [35]. Australia’s commitment to action was published almost a decade before Canada’s Truth and Reconciliation Commission’s Final Report and Calls to Action, which may explain why an overwhelming majority of studies selected for this review were published in Australia—nearly 2.5 times the number of studies published in Canada, and more than three times the number published in the United States. 

### 5.2. Limitations

The main limitation of this study is that the data presented is somewhat dated. The article searches were undertaken in mid-2020, in the early stages of the COVID-19 pandemic, and with no dedicated research funding. New priorities to understand and address Indigenous peoples’ experiences with the COVID-19 became competing priorities with this study, and at a time when there was increased demand for Indigenous CST. As a result, our team used the preliminary findings from this review to prioritize the development of Oshki M’naadendimowin, a 36 h Indigenous cultural safety training program for faculty members, staff, and students at the Faculties of Medicine, Nursing, Social Work, and Public Health at the University of Toronto. For these reasons, combined with the large number of articles and data categories included in this review, we were not able to update the article searches to 2023. There is also the possibility that some relevant studies may have been missed due to the selection of databases or the exclusion of grey literature. The language skills of the researchers on the team also limited the searches to English publications, where French, Spanish, or Indigenous languages may have been used within the selected geographic regions. Furthermore, search terms for populations did not include Indigenous nation-specific names (e.g., Mohawk, Cree), and thus publications that did not use overarching terms such as Indigenous or First Nations may have been missed.

## 6. Conclusions

This review presents the substantial work undertaken over the last three decades in Canada, Australia, New Zealand, and the United States to deliver Indigenous cultural safety training interventions to address anti-Indigenous racism and its historical legacy in national, publicly funded health and education systems. While the research findings from this review set out the substantive progress toward developing training interventions, this field of research remains in its infancy. Future research on cultural safety and related training interventions requires greater clarity in conceptualization of cultural related concepts, and more robust approaches to evaluate such interventions and identify which training strategies are most effective. Moreover, future work is required to identify how and when cultural safety and related concepts should be applied, and this is likely to vary across countries, regions, and relevant Indigenous groups.

Finally, to advance future research and practice on Indigenous cultural safety, relevant Indigenous groups must be intentionally and meaningfully engaged throughout the entire duration of research and practice. A 2021 report published by the Yellowhead Institute on Canada’s progress toward reconciliation concluded that symbolic actions were being prioritized by the Canadian government over lasting permanent and structural changes necessary to transform the country’s relationship with Indigenous peoples [36]. It is imperative that current momentum to advance the field on Indigenous cultural safety training is capitalized upon to address anti-Indigenous racism and the legacy that colonial governments continue to impose upon Indigenous peoples in counties such as Canada.

## Figures and Tables

**Table 1:** Search Strategy.

Subject Heading Search		
Culturally Competent Care OR Cultural Competency OR Cultural Competence OR Cultural Safety OR Cultural Sensitivity AND Indigenous Peoples OR American Native Continental Ancestry Group OR Oceanic Ancestry Group AND Health Personnel OR Education OR Curriculum OR Teaching OR Social Work OR Social Work Service OR Students, Social Work OR Education, Social Work OR Social Service Assessment OR Facilities, Manpower and Services OR Occupational Health Services		
Keyword Search		
Search categories		Search terms
Activity	Indigenous cultural Safety Training	“cultural safety” OR “cultural competence” OR “culturally appropriate” OR “cultural sensitivity” AND
Context	Indigenous	Indigenous OR “First Nations” OR “Métis” OR “Inuit” OR “Aboriginal” OR “Maori” OR “Torres Straight” AND
Population	Health	“health care” OR “practitioner” OR “health care provider” OR “health professional” OR nurse OR physician OR “public health” AND
	Education	“education” OR “teacher” OR “faculty” OR “curriculum” AND
	Social Services	“social work” OR “child welfare” OR “criminal justice work” OR “support work” OR “employment support” OR “housing service” OR “family service” or “child aid” OR “child service” OR “youth service”
Databases selected		Medline, Embase, CINAHL, ERIC, and ASSIA

**Table 2:** General Characteristics of Sources of Evidence.

General Characteristics	Number (*n* = 134)	Percent (%)
Publication Year		
2011–2020	93	69%
2001–2010	35	26%
1996–2000	6	5%
Country(ies) of Study *		
Australia	66	49%
Canada	28	21%
United States	21	16%
New Zealand	20	15%
Author(s) Self-identified as Indigenous		
Yes	26	20%
Relevant Indigenous Group Specified in Article		
Aboriginal (Australia)	66	49%
Torres Strait Islander Australia)	46	34%
Māori/Tangata Whenua	20	15%
First Nation (Canada)	18	13%
American Indian (United States)	17	12%
Métis (Canada)	12	9%
Inuit (Canada)	11	8%
Unspecified (Canada)	10	7%
Alaska Native (United States)	6	4%
Kānaka Maoli/Native Hawai’ians (United States)	2	1%
Unspecified (United States)	2	2%
Target Discipline(s)/Field(s)		
Nursing/midwifery	45	34%
General health ^±^	31	23%
Medicine/physicians	25	19%
Allied health ^¥^	18	13%
Social work/services	15	11%
Education ^α^	13	10%
Target Population(s)		
Students only	64	48%
Professionals/academics only	62	46%
Professionals/academics and students	8	6%
Cultural Safety (or similar) Concepts and Critiques		
Described and/or cited a cultural safety (or related) concept	69	51%
Described and/or cited a critique of a cultural safety (or related) concept	26	19%
Cultural Safety Training Interventions and Evaluations of Interventions		
Described an Indigenous cultural safety training Intervention	69	51%
Described an evaluation of an Indigenous cultural safety training intervention	61	46%

* A study undertaken in both Canada and the United States was counted twice. ^±^ General Health refers to Public Health professionals, Psychologists, health researchers, and otherwise unspecified health professionals or practitioners. ^¥^ Allied Health Professionals refers to Dentists, Pharmacists, Occupational Therapists, Physiotherapists, Audiologists and Speech Language Therapists, and Radiologists. ^α^ Education refers both to those in the field of Education and to university students/staff in unspecified fields.

**Table 3:** Defining Cultural Safety, Cultural Competence, and Similar Concepts (Baba 2013) [34].

Concept	Definition
Cultural Awareness	The acknowledgement and understanding of cultural differences by focusing on the “other” and the “other culture”. Does not consider the political or social-economic influences on cultural difference and does not require an individual to reflect on his/her own cultural perspectives.
Cultural Sensitivity	Recognizes the need to respect cultural differences. Involves exhibiting behaviours that are considered polite and respectful by the persons of other cultures. Focuses on the “other” and the “other culture” and does not require an individual to reflect on his/her own cultural perspectives.
Cultural Competence	Skills and behaviours that help a practitioner provide quality care to diverse populations. While cultural competence can build upon self-awareness, it is limited by reducing culture to a set of skills for practitioners to master and over-emphasizes cultural differences as the course of conflict between healthcare providers and diverse populations.
Cultural Safety	Cultural safety within an Indigenous context means that the educator, practitioner, or professional, whether Indigenous or not, can communicate competently with a patient in that patient’s social, political, linguistic, economic, and spiritual realm. Moves beyond cultural sensitivity to analyzing power imbalances, institutional discrimination, colonization, and colonial relationships as they apply to healthcare.

## Data Availability

A Preprint of this manuscript is available online via BioRxiv (www.borxiv.org, accepted on 7 October 2022). https://doi.org/10.1101/2022.10.06.511097.

## Appendix A

**Table A1:** Data Themes and Focus.

Data Themes	Focus
Article Characteristics	Title Journal Publication Year Country of Study Authors Author(s) self-identified as Indigenous: [yes OR no] Study aim/objective(s) Target Field(s)/Discipline(s): [Medicine; Nursing/Midwifery; Allied Health; Health, General; Education; Social Work] Target Population: [students; professionals]
2. Cultural Safety Concepts, Critiques, and Rationale	Articulate/cite a cultural safety or related concept Critique a cultural safety or related concept Rationale for cultural safety
3. Characteristics of Cultural Safety Training (if applicable)	Article described a cultural safety training? [yes OR no] [If yes, continue below]: Who sponsored the training? Who developed the training? Is there evidence of Indigenous scholar, practitioner, knowledge keeper or community members in the development of the training [yes OR no] Which Indigenous community partners were engaged? Who delivered the training? No. years training has run (r/t publication year) Who received the training [Profession; Setting (rural OR urban)]? Training objectives Modalities used to deliver the training Training duration/timeline Brief description of training components Number of people who completed the training Details of post training supports provided Stated limitations of the training
4. Evaluation Details of Cultural Safety Training (if applicable)	Did article present an evaluation of a cultural safety training? [yes OR no] [If yes, continue below]: Evaluation objective(s) Evaluation data collection methods Type of data collected (qualitative; quantitative) Key evaluation results Stated limitations of evaluation Recommendations provided Was a data collection tool provided [yes OR no]

## Appendix B

**Table A2:** Reference List of Sources Included in Scoping Review.

Papps E, Ramsden I. Cultural safety in nursing: The New Zealand experience. Int J Qual Health Care. 1996, 491–497. Cunningham J, Wollin J. The importance of clinical experience in aboriginal communities. Contemp Nurse. 1998, 7, 152–155. Polaschek N. Cultural safety: A new concept in nursing people of different ethnicities. Journal of Advanced Nursing. 1998, 22, 452–457. Weaver HN. Indigenous people in a multicultural society: Unique issues for human services. Social Work 1998; 43: 203–211. Weaver HN. Indigenous people and the social work profession: Defining culturally competent services. Social Work. 1999; 44: e1–e18 Weaver HN. Transcultural nursing with Native Americans: Critical knowledge, skills, and attitudes. Journal of Transcultural Nursing. 1999; 10: 197–202. Palafox NA, Buenconsejo-Lum L, Ka’Ano’I M, Yamada S. Cultural competence: A proposal for physicians reaching out to Native Hawaiian patients. Pacific Health Dialogue. 2001; 8: 388–392. Allen J. Assessment training for practice in American Indian and Alaska Native settings. J Pers Assess. 2002; 79: 216–225. Richardson S. Aoteaoroa New Zealand nursing: From eugenics to cultural safety. Nursing Inquiry. 2004; 11: 35–42. Hurt DA, Michael WL. Teaching American Indian geography and history with new perspectives: The Lodge Pole River Project example. Journal of Geography. 2005; 104: 187–193. Langer CL. The effect of selected macro forces on the contemporary social construction of American Indian ethnic identity. Journal of Health and Social Policy. 2005; 20: 15–32. Spence DG. Hermeneutic Notions Argument Cultural Safety Education. Journal of Nursing Education. 2005; 44: 409–414. Browne AJ, Varcoe C. Critical cultural perspectives and health care involving Aboriginal peoples. Contemp Nurse. 2006; 22: 155–167. Fredericks B. Which way? Educating for nursing Aboriginal and Torres Strait Islander peoples. Contemp Nurse. 2006; 23: 87–99. Horsburgh M, Merry A, Seddon M, Baker H, Poole P, Shaw J, et al. Educating for healthcare quality improvement in an interprofessional learning environment: A New Zealand initiative. J Interprof Care. 2006; 20: 555–557. Kruske S, Kildea S, Barclay L. Cultural safety and maternity care for Aboriginal and Torres Strait Islander Australians. Women Birth. 2006; 19: 73–77. Main C, McCallin A, Smith N. Cultural safety and cultural competence what does this mean for physiotherapists. NZ Journal of Physiotherapy. 2006; 34: 160–166. Morgan S. Orientation for general practice in remote Aboriginal communities: A program for registrars in the Northern Territory. Aust J Rural Health. 2006; 14: 202–208. Nash R, Meiklejohn B, Sacre S. The Yapunyah project: Embedding Aboriginal and Torres Strait Islander perspectives in the nursing curriculum. Contemp Nurse. 2006; 22: 296–316. Ratima M, Waetford C, Wikaire E. Cultural competence for physiotherapists: reducing inequalities in health between Maori, non-Maori. NZ Journal of Physiotherapy. 2006; 34: 153–159. Bazen J, Paul D, Tennant M. An aboriginal and Torres Strait Islander oral health curriculum framework development experiences in W Australia. Australian Dental Journal. 2007; 52: 86–92. Johnstone MJ, Kanitsaki O. An exploration of the notion and nature of the construct of cultural safety and its applicability to the Australian health care context. J Transcult Nurs. 2007; 18: 247–256. Kana P, Normore AH, Aitken V. “She didn’t ask me about my grandma”. Journal of Educational Administration. 2007; 45(6): 697–710. Richardson S, Williams R. Why is cultural safety essential in health care? Med Law. 2007; 26: 699–707. Amundson ML, Moulton PL, Zimmerman SS, Johnson BJ. An innovative approach to student internships on American Indian reservations. J Interprof Care. 2008; 22: 93–101. Begoray D, Banister E. Learning about Aboriginal contexts: The reading circle approach. Journal of Nursing Education. 2008; 47: 324–326. DeSouza R. Wellness for all: The possibilities of cultural safety and cultural competence in New Zealand. Journal of Research in Nursing. 2008; 13: 125–135. Pinnock R, Jones R, Wearn A. Learning and assessing cultural competence in paediatrics. Med Educ. 2008; 42: 1111–1146. Ranzijn R, McConnochie K, Day A, Nolan W, Wharton M. Towards cultural competence: Australian Indigenous content in undergraduate psychology. Australian Psychologist. 2008; 43: 132–139. Abuzar MA, Burrow MF, Morgan M. Development of a rural outplacement programme for dental undergraduates: Students’ perceptions. Eur J Dent Educ. 2009; 13: 233–239. Andersen C. Indigenous footprints on health curriculum. The Australian Journal of Indigenous Education. 2009; 38: 40–45. Browne AJ, Varcoe C, Smye V, Reimer-Kirkham S, Lynam JM, Wong S. Cultural safety and the challenges of translating critically oriented knowledge in practice. Nursing Philosophy. 2009; 10: 167–179. Charbonneau CC, Nurfeld MJ, Craig BJ, Donnelly L, R. Increasing cultural competence in the dental hygiene profession. Can J Dent Hygiene. 2009; 43: 297–305. Mays VE, Gallardo M, Shorter-Gooden K, Robinson-Zanartue C, Smith M, McClure F, et al. Expanding the circle: Decreasing American Indian mental health disparities through culturally competent teaching about American Indian mental health. Am Indian Cult Res. 2009; 33: 61–83. Nagel T, Thompson C, Spencer N, Judd J, Williams R. Two way approaches to Indigenous mental health training brief training in brief interventions. Australian e-Journal for the Advancement of Mental Health. 2009; 8: 1–7. Ambtman R, Hudson S, Hartry R, MacKay-Chiddenton D. Promoting system-wide cultural competence for serving Aboriginal families and children in a midsized Canadian city. Journal of Ethnic And Cultural Diversity in Social Work. 2010; 19: 235–251. Anning B. Embedding an Indigenous graduate attribute into University of Western Sydney’s courses. The Australian Journal of Indigenous Education. 2010; 39(S1): 40–52. Durey A. Reducing racism in Aboriginal health care in Australia: Where does cultural education fit? Aust N Z J Public Health. 2010; 34: S87–S92. Evans I, Fitzgerald J, Herbert A, Harvey S. Cultural competence and power sharing: An international perspective on training clinical child psychologists. The Journal of Mental Health Training, Education and Practice. 2010; 5: 34–42. Jones R, Pitama SG, Huria T, Poole P, McKimm J, Pinnock R, et al. Medical education to improve Maori health. The New Zealand Medical Journal 2010; 123: 113–139. Phiri J, Dietsch E, Bonner A. Cultural safety and its importance for Australian midwifery practice. Collegian. 2010; 17: 105–111. Bernhardt BM, Green E, Khurana A, Laporte T, Osmond S, Panchyk H, et al. Course development at the University of British Columbia concerning audiology and speech-language pathology for people of First Nations, Métis, and Inuit Heritage. Canadian Journal of Speech-Language Pathology and Audiology. 2011; 35: 178–189. Blackman R. Understanding culture in practice: Reflections of an Australian Indigenous nurse. Contemporary Nurse. 2011; 37: 31–34. Bourque Bearskin RL. A critical lens on culture in nursing practice. Nursing Ethics. 2011; 18: 548–559. Crow K, Conger MM, Knoki-Wilson U. Mentorship for nursing in a rural area: A program for success for working with diverse populations. Online Journal of Rural Nursing and Health Care. 2011; 11: 43–50. Downing R, Kowal E. Putting Indigenous cultural training into nursing practice. Contemp Nurse. 2011; 37: 10–20. Mahara MS, Duncan SM, Whyte N, Brown J. It takes a community to raise a nurse: Educating for culturally safe practice with Aboriginal peoples. International Journal of Nursing Education Scholarship. 2011; 8: 1–12. Moosa T, Schurr S. Reflections on a Northern Ontario placement initiative. Canadian Journal of Speech-Language Pathology and Audiology. 2011; 35: 160–167. Zeidler D. Building a relationship: Perspectives from one First Nations community. Canadian Journal of Speech-Language Pathology and Audiology. 2011; 35: 136–143. Zinck K, Marmion S. Global focus, local acts: Providing mental health services to Indigenous people. Arch Psychiatr Nurs. 2011; 25(5): 311–319. Abu Rass R. Supporting newly recruited teachers in a unique area, the Northwest Territories in Canada. Journal of Education for Teaching. 2012; 38: 141–161. Doutrich D, Arcus K, Dekker L, Spuck J, Pollock-Robinson C. Cultural safety in New Zealand and the United States: Looking at a way forward together. J Transcult Nurs. 2012; 23: 143–150. Gerlach AJ. A critical reflection on the concept of cultural safety. Can J Occup Ther. 2012; 79: 151–158. Harrison EB, Lautensach AK, McDonald VL. Moving from the margins: Culturally safe teacher education in remote northwestern British Columbia. McGill Journal of Education. 2012; 47: 323–343. Martin T, DiRienza M. Closing the gap in a regional health service in NSW: A multi-strategic approach to addressing racism. NSW Public Health Bulletin. 2012; 23: 63–67. Oda K, Rameka M. Students’ corner: Using Te Tiriti O Waitangi to identify and address racism, and achieve cultural safety in nursing. Contemp Nurse. 2012; 43: 107–112. Wendt DC, Gone JP. Rethinking cultural competence: insights from indigenous community treatment settings. Transcult Psychiatry. 2012; 49: 206–222. Flavell H, Thackrah R, Hoffman J. Developing Indigenous Australian cultural competence: A model for implementing Indigenous content. Journal of Teaching and Learning for Graduate Employability. 2013; 4: 39–63. Goerke V, Kickett M. Working towards the assurance of graduate attributes for ICS Training. The International Education Journal: Comparative Perspectives. 2013; 12: 61–81. Herring S, Spangaro J, Lauw M, McNamara L. The intersection of trauma, racism, and cultural competence in Effective work with Aboriginal people: Waiting for trust. Australian Social Work. 2013; 66: 104–117. Jackson D, Power T, Sherwood J, Geia L. Amazingly resilient Indigenous people! Using transformative learning to facilitate positive student engagement with sensitive material. Contemp Nurse. 2013; 46: 105–112. Rowan MS, Rukholm E, Bourque-Bearskin L, Baker C, Voyageur E, Robitaille A. Cultural competence and cultural safety in Canadian schools of nursing: A mixed methods study. Int J Nurs Educ Scholarsh. 2013; e1–10. Thackrah RD, Thompson SC. Confronting uncomfortable truths: Receptivity and resistance to Aboriginal content in midwifery education. Contemp Nurse. 2013; 46: 113–122. Thackrah RD, Thompson SC. "Friendly racism" and white guilt: Midwifery students’ engagement with Aboriginal content in their program. Forum on Public Policy: a journal of the Oxford Round Table. 2013; e1–12. Abbott P. What do GPs need to work more effectively with Aboriginal patients? Views of Aboriginal cultural mentors and health workers. Australian Family Physician. 2014; 43: 58–63. Chapman R, Martin C, Smith T. Evaluation of staff cultural awareness before and after attending cultural awareness training in an Australian emergency department. Int Emerg Nurs. 2014; 22: 179–184. Chiodo LN, Sonn CC, Morda R. Implementing an intercultural psychology undergraduate unit: Approach, strategies, and outcomes. Australian Psychologist. 2014; 49: 181–192. Durey A, Wynaden D, O’Kane M. Improving forensic mental health care to Indigenous Australians: Theorizing the intercultural space. J Psychiatr Ment Health Nurs. 2014; 21: 296–302. Ferdinand AS, Paradies Y, Perry R, Kelaher M. Aboriginal health promotion through addressing employment discrimination. Aust J Prim Health. 2014; 20: 384–388. Hudson G, Maar M. Faculty analysis of distributed medical education in Northern Canadian Aboriginal communities. Rural and Remote Health. 2014; 14: 1–8. Jacklin K, Strasser R, Peltier I. From the community to the classroom: The Aboriginal health curriculum at the North Ontario School of Medicine. Can J Rural Med. 2014; 19: 143–150. Kickett M, Hoffman J, Flavell H. A model for large-scale, interprofessional, compulsory cross-cultural education with an indigenous focus. Journal of Allied Health. 2014; 43: 38–44. Mlcek S. Are we doing enough to develop cross-cultural competencies for social work? British Journal of Social Work. 2014; 44: 1984–2003. Averill R, Anderson D, Drake M. Developing culturally responsive teaching through professional noticing within teacher educator modelling. Mathematics Teacher Education and Development. 2015; 17: 64–83. Beavis AS, Hojjati A, Kassam A, Choudhury D, Fraser M, Masching R, et al. What all students in healthcare training programs should learn to increase health equity: perspectives on postcolonialism and the health of Aboriginal peoples in Canada. BMC Med Educ. 2015; 15: e1–11. Bennet M, Moriarty B. Language, relationships, and pedagogical practices: pre-service teachers in an Indigenous Australian context. International Journal of Pedagogies and Learning. 2015; 10: e1–13. Carey M. The limits of cultural competence: An Indigenous Studies perspective. Higher Education Research & Development. 2015; 34: 828–840. Doucette H, Maillet PJ, Brillant MG, Tax CL. Dental hygiene students’ perceptions of a cultural competence component in a tobacco dependence education curriculum a pilot study. Allied Dental Education. 2015; 79: 680–685. Hart B, Cavanagh M, Douglas D. The “Strengthening Nursing Culture Project”—an exploratory evaluation study of nursing students’ placements within Aboriginal Medical Services. Contemp Nurse. 2015; 51: 245–256. Hunt L, Ramjan L, McDonald G, Koch J, Baird D, Salamonson Y. Nursing students’ perspectives of the health and healthcare issues of Australian Indigenous people. Nurse Educ Today. 2015; 35: 461–467. Liaw ST, Hasan I, Wade V, Canalese R, Kelaher M, Lau P, et al. Improving cultural respect to improve Aboriginal health in general practice multi-methods study. Australian Family Physician. 2015; 44: 387–392. Patel B. Communicating across cultures: Proceedings of a workshop to assess health literacy and cross-cultural communication skills. Journal of Pharmacy Practice and Research. 2015; 45: 49–56. Pridham B, Martin D, Walker K, Rosengren R, Wadley D. Culturally inclusive curriculum in higher education. The Australian Journal of Indigenous Education. 2015; 44: 94–105. Riley L, Howard-Wagner D, Mooney J. Kinship Online: Engaging ‘cultural praxis’ in a teaching and learning framework for cultural competence. The Australian Journal of Indigenous Education. 2015; 44: 70–84. Smith J, Wolfe CL, Springer S, Martin M, Togno J. Using cultural immersion as the platform for teaching Aboriginal Torres Strait Islander health in an undergraduate medical curriculum. Rural and Remote Health. 2015; 15: e1–9. Tipa Z, Wilson D, Neville S, Adams J. Cultural responsiveness and the family partnership model. Nursing Praxis in New Zealand. 2015; 31: 35–49. Bennet M, Moriarty B. Lifelong learning theory and pre-service teachers’ development of knowledge and dispositions to work with Australian Aboriginal students. International Journal of Pedagogies and Learning. 2016; 11: e1–9. Brewer KM, McCann CM, Harwood MLN. The complexities of designing therapy for Māori living with stroke-related communication disorders. New Zealand Medical Journal. 2016; 129: 75–82. Browne AJ, Varcoe C, Lavoie J, Smye V, Wong ST, Krause M, et al. Enhancing health care equity with Indigenous populations: evidence-based strategies from an ethnographic study. BMC Health Serv Res. 2016; 16: e1–17. Coke S, Kuper A, Richardson L, Cameron A. Northern perspectives on medical elective tourism: A qualitative study. CMAJ Open. 2016; 4: E277–E283. Dell EM, Firestone M, Smylie J, Vaillancourt S. Cultural safety and providing care to Aboriginal patients in the Emergency Department. CJEM. 2016; 18: 301–305. Isaacs AN, Raymond A, Jacob E, Jones J, McGrail M, Drysdale M. Cultural desire need not improve with cultural knowledge: A cross-sectional study of student nurses. Nurse Educ Pract. 2016; 19: 91–96. Jacob E, Raymond A, Jones J, Jacob A, Drysdale M, Isaacs AN. Exploration of nursing degree students’ content expectations of a dedicated Indigenous health unit. Collegian. 2016; 23: 313–319. Kelly J, Wilden C, Chamney M, Martin G, Herman K, Russell C. Improving cultural and clinical competency and safety of renal nurse education. Renal Society of Australasia Journal. 2016; 12: 106–112. Power T, Virdun C, Sherwood J, Parker N, Van Balen J, Gray J, et al. REM: A collaborative framework for building Indigenous cultural competence. J Transcult Nurs. 2016; 27: 439–446. Coombe L, Lee V, Robinson P. Integration models for indigenous public health curricula. Higher Education Research & Development. 2017; 36: 645–659. Darroch F, Giles A, Sanderson P, Brooks-Cleator L, Schwartz A, Joseph D, et al. The United States does CAIR about cultural safety: Examining cultural safety within Indigenous health contexts in Canada and the United States. J Transcult Nurs. 2017; 28: 269–277. Durey A, Halkett G, Berg M, Lester L, Kickett M. Does one workshop on respecting cultural differences increase health professionals’ confidence to improve the care of Australian Aboriginal patients with cancer? An evaluation. BMC Health Serv Res. 2017; 17: e1–13. Fleming T, Creedy DK, West R. Impact of a continuing professional development intervention on midwifery academics’ awareness of cultural safety. Women Birth. 2017; 30: 245–252. Forsyth C, Irving M, Tennant M, Short S, Gilroy J. Indigenous cultural competence: A dental faculty curriculum review. Eur J Dent Educ. 2017; 22: e419–e426. Herzog LS. The need for narrative reflection and experiential learning in medical education: A lesson learned through an urban indigenous health elective. Med Teach. 2017; 39: 995–996. Huria T, Palmer S, Beckert L, Lacey C, Pitama S. Indigenous health: designing a clinical orientation program valued by learners. BMC Med Educ. 2017; 17: e1–8. Jameison M, Chen S, Murphy S, Maracle L, Mofina A, Hill J. Pilot testing an intervention on cultural safety and Indigenous health in a Canadian occupational therapy curriculum. Journal of Allied Health. 2017; 46: e1–7. Wilson S, McChesney J, Brown L. Cultural competencies and planning for teaching math: Preservice teachers responding to expectations, opportunities, and resources. Journal of Urban Mathematics Education. 2017; 10: 95–112. Alexander-Ruff JH, Kinion E. Engaging nursing students in a rural Native American community to facilitate cultural consciousness. J Community Health Nurs. 2018; 35: 196–206. Battersby M, Lawn S, Kowanko I, Bertossa S, Trowbridge C, Liddicoat R. Chronic condition self-management support for Aboriginal people: Adapting tools and training. Aust J Rural Health. 2018; 26: 232–237 Bennett B, Redfern H, Zubrzycki J. Cultural responsiveness in action: Co-constructing social work curriculum resources with Aboriginal communities. The British Journal of Social Work. 2018; 48: 808–825. Bolton J, Andrews S. "I learned more than from any lecture"—Indigenous place and space for teaching Indigenous health to physiotherapy students. Physical Therapy Reviews. 2018; 23: 35–39. Browne AJ, Varcoe C, Ford-Gilboe M, Nadine Wathen C, Smye V, Jackson BE, et al. Disruption as opportunity: Impacts of an organizational health equity intervention in primary care clinics. Int J Equity Health. 2018; 17: e1–16. Crowshoe LL, Han H, Calam B, Henderson R, Jacklin K, Walker L, et al. Impacts of educating for equity workshop on addressing social barriers of type 2 diabetes with Indigenous patients. J Contin Educ Health Prof. 2018; 38: 49–59. Cueva K, Revels L, Cueva M, Lanier AP, Dignan M, Viswanath K, et al. Culturally-relevant online cancer education modules empower Alaska’s community health aides/practitioners to disseminate cancer information and reduce cancer risk. J Cancer Educ. 2018; 33: 1102–1109. Currie G, Wheat J, Wess T. Building foundations for Indigenous cultural competence: An institution’s journey toward “Closing the Gap”. J Med Imaging Radiat Sci. 2018; 49: 6–10. Johnson R, Withers M. Cultural competence in the emergency department: Clinicians as cultural learners. Emerg Med Australas. 2018; 30(6): 854–856. Kreif T, Yoshimotot YS, Mokuau N. Ke A’o Mau strengthening cultural competency in interdisciplinary education. Hawai’i Journal of Medicine & Public Health. 2018; 77: 333–336. Lavery S, Cain G, Hampton P. Walk beside me, learn together: A service-learning immersion to a remote Aboriginal school and community. Australian and International Journal of Rural Education. 2018; 28: e1–16. Lewis ME, Hartwell EE, Myhra LL. Decolonizing mental health services for Indigenous clients: A training program for mental health professionals. Am J Community Psychol. 2018; 62: 330–339. Thackrah RD, Thompson SC. Applying a midwifery lens to Indigenous health care delivery: The contribution of campus learning and rural placements to effecting systemic change. Can J Nurs Res. 2018; 50: 179–188. Bennett B, Gates TG. Teaching cultural humility for social workers serving LGBTQI Aboriginal communities in Australia. Social Work Education. 2019; 38: 604–617. Duthie D. Embedding Indigenous knowledges and cultural safety in social work curricula. Australian Social Work. 2019; 72: 113–116. Fitzpatrick SA, Haswell MR, Williams MM, Nathan S, Meyer L, Ritchie JE, et al. Learning about Aboriginal health and wellbeing at the postgraduate level: Novel application of the Growth and Empowerment Measure. Rural Remote Health. 2019; 19: e1–13. Forsyth C, Irving M, Short S, Tennant M, Gilroy J. Strengthening Indigenous cultural competence in dentistry and oral health education: Academic perspectives. Eur J Dent Educ. 2019; 23: e37–44. Gadsden T, Wilson G, Totterdell J, Willis J, Gupta A, Chong A, et al. Can a continuous quality improvement program create culturally safe emergency departments for Aboriginal people in Australia? A multiple baseline study. BMC Health Serv Res. 2019; 19: e1–15. Garcia AN, Castro MC, Sanchez JP. Social and structural determinants of urban American Indian and Alaska Native health: A case study in Los Angeles. MedEdPORTAL. 2019; 15: e1–9. Haight W, Waubanascum C, Glesener D, Day P, Bussey B, Nichols K. The Center for Regional and Tribal Child Welfare Studies: Reducing disparities through indigenous social work education. Children and Youth Services Review. 2019; 100: 156–166. Liaw ST, Wade V, Furler JS, Hasan I, Lau P, Kelaher M, et al. Cultural respect in general practice: A cluster randomised controlled trial. Med J Aust. 2019; 210: 263–268. McMichael B, Nickel A, Duffy EA, Skjefte L, Lee L, Park P, et al. The impact of health equity coaching on patient’s perceptions of cultural competency and communication in a pediatric emergency department: An intervention design. J Patient Exp. 2019; 6: 257–264. Muise GM. Enabling cultural safety in Indigenous primary healthcare. Healthc Manage Forum. 2019; 32(1): 25–31. Parker M, Pearson C, Donald C, Fisher CB. Beyond the Belmont Principles: A community-based approach to developing an Indigenous ethics model and curriculum for training health researchers working with American Indian and Alaska Native communities. Am J Community Psychol. 2019; 64: 9–20. Zimmerman P-A, Stringfellow T, Rowland D, Armstrong V, West R. Review of Aboriginal and Torres Strait Islander content within a Bachelor of Nursing. Collegian. 2019; 26: 441–447. Fleming T, Creedy DK, West R. The influence of yarning circles: A cultural safety professional development program for midwives. Women Birth. 2020; 33: 175–185. Forsyth C, Short S, Gilroy J, Tennant M, Irving M. An Indigenous cultural competence model for dentistry education. Br Dent J. 2020; 228: 719–725. Harris T, O’Donoghue K. Developing culturally responsive supervision through yarn up time and the CASE supervision model. Australian Social Work. 2020; 73: 64–76. Kanji Z, Lin D, Karan J. Assessing dental hygiene students’ readiness for interprofessional learning and collaborative practice. J Dent Educ. 2020; 84: 669–680. Sunderland N, Woods G, Dorsett P. Making the invisible visible: Applying digital storytelling for immersive, transformative, and anti-colonial learning. The British Journal of Social Work. 2020; 50: 483–505.

## Appendix C

**Table A3:** Citations for General Characteristics of Sources of Evidence.

General Characteristics		Sources
Publication Year		
	2011–2020	[37,38,39,40,41,42,43,44,45,46,47,48,49,50,51,52,53,54,55,56,57,58,59,60,61,62,63,64,65,66,67,68,69,70,71,72,73,74,75,76,77,78,79,80,81,82,83,84,85,86,87,88,89,90,91,92,93,94,95,96,97,98,99,100,101,102,103,104,105,106,107,108,109,110,111,112,113,114,115,116,117,118,119,120,121,122,123,124,125,126,127]
	2001–2010	[25,33,128,129,130,131,132,133,134,135,136,137,138,139,140,141,142,143,144,145,146,147,148,149,150,151,152,153,154,155,156,157,158,159,160,161,162]
	1996–2000	[28,163,164,165,166,167]
Country(ies) of Study *		
	Australia	[33,44,45,46,47,54,55,57,61,64,66,67,68,72,74,75,76,77,79,80,83,85,86,88,90,91,93,94,95,97,98,99,102,103,106,107,109,110,111,112,113,114,115,116,118,121,123,124,125,126,127,129,130,131,134,137,140,143,144,147,150,151,155,158,159,167]
	Canada	[39,40,41,43,50,51,52,53,58,60,63,70,71,78,82,89,92,104,108,117,119,120,122,135,139,142,152,161].
	United States	[38,48,49,56,69,71,73,84,87,100,101,105,128,133,141,153,160,162,163,165,166]
	New Zealand	[25,28,37,42,59,62,65,81,96,132,136,138,145,146,148,149,154,156,157,164]
Author(s) Self-identified as Indigenous		
	Yes	[28,38,40,42,46,49,53,55,57,63,67,70,72,74,75,79,85,87,88,91,101,112,120,126,134,143]
Relevant Indigenous Group Specified in Article		
	Aboriginal (Australia)	[33,44,45,46,47,54,55,57,61,64,66,67,68,72,74,75,76,77,79,80,83,85,86,88,90,91,93,94,95,97,98,99,102,103,106,107,109,110,111,112,113,114,115,116,118,121,123,124,125,126,127,129,130,131,134,137,140,143,144,147,150,151,155,158,159,167]
	Torres Strait Islander (Australia)	[33,44,45,46,47,54,55,57,61,64,68,74,75,76,77,79,80,88,91,93,94,95,97,106,111,112,113,114,115,116,118,121,123,124,126,129,130,131,134,137,140,144,147,155,158,159]
	Māori/Tangata Whenua	[25,28,37,42,59,62,65,81,96,132,136,138,145,146,148,149,154,156,157,164]
	First Nation (Canada)	[39,40,41,43,50,51,52,53,58,63,78,92,105,108,117,120,122,161]
	American Indian (United States)	[38,49,56,71,73,87,100,101,105,128,141,153,160,162,163,165,166]
	Métis (Canada)	[39,40,52,58,60,70,71,117,120,122,142,161]
	Inuit (Canada)	[39,40,52,58,60,70,71,92,117,120,142]
	Unspecified (Canada)	[51,53,63,82,89,104,119,135,139,152]
	Alaska Native (United States)	[38,69,71,101,128,166]
	Kānaka Maoli/Native Hawai’ians (United States)	[84,133]
	Unspecified (United States)	[48,87]
Target Discipline(s)/Field(s)		
	Nursing/midwifery	[25,28,33,47,49,50,54,55,56,57,59,60,61,62,63,71,80,88,93,95,96,97,98,99,100,118,119,121,129,131,132,134,135,137,138,139,140,142,143,154,156,164,165,167]
	General health ^±^	[38,44,45,46,52,58,69,71,84,85,87,93,94,103,105,112,115,118,119,123,124,125,128,129,130,136,141,144,146,155,158,160]
	Medicine/physicians	[25,49,51,53,55,64,65,68,70,71,84,101,102,104,116,117,119,120,122,127,129,132,133,150,157]
	Allied health ^¥^	[25,39,40,77,78,79,81,82,89,108,109,118,129,145,147,148,151,152]
	Social work/services	[25,39,40,77,78,79,81,82,89,108,109,118,129,145,147,148,151,152]
	Education ^α^	[37,41,42,43,86,90,91,110,111,126,149,153,159]
Target Population(s)		
	Students only	[25,28,37,40,42,47,50,51,52,54,57,60,65,67,72,73,74,75,76,78,79,81,82,83,84,85,86,89,91,92,93,94,95,97,99,100,103,106,107,109,110,111,112,114,115,116,117,120,121,122,126,129,131,132,142,144,146,147,149,151,157,159,160,167]
	Professionals/academics only	[33,38,39,43,44,45,46,48,49,53,55,58,59,61,62,63,64,66,68,69,70,71,77,80,87,88,90,96,102,104,105,108,113,118,119,123,124,125,128,130,134,135,136,137,138,139,140,143,145,148,150,152,153,154,155,156,161,162,163,164,165,166]
	Professionals/academics and students	[41,56,98,101,127,133,141,158]
Cultural Safety (or similar) Concepts and Critiques		
	Described and/or cited a cultural safety (or related) concept	[28,33,39,40,41,42,46,47,52,53,54,55,57,58,59,60,61,62,63,67,71,72,76,79,80,87,90,92,93,94,96,97,98,100,102,104,105,108,110,114,115,117,118,119,121,122,124,129,130,131,133,134,136,137,138,139,143,145,146,148,151,152,154,156,157,158,161,164,166]
	Described and/or cited a critique of a cultural safety (or related) concept	[28,33,39,46,52,58,61,62,63,67,71,87,93,105,114,115,121,130,135,137,138,139,154,157,158,164]
Cultural Safety Training Interventions and Evaluations of Interventions		
	Described an Indigenous cultural safety training Intervention	[25,40,42,43,44,45,47,49,50,51,55,56,57,65,66,68,70,73,74,76,77,78,80,82,83,84,85,86,87,88,89,91,92,93,94,97,99,100,101,103,109,110,111,112,113,114,115,116,118,119,121,122,125,127,129,131,132,141,142,144,147,149,150,151,153,155,160,161,167]
	Described an evaluation of an Indigenous cultural safety training intervention	[40,42,43,47,49,51,54,55,57,59,62,64,65,66,68,69,70,72,73,74,78,80,82,84,85,86,88,89,92,94,97,99,100,101,103,106,107,109,110,111,112,113,114,115,116,118,119,121,125,131,132,140,141,144,151,153,155,160,161,163,165]

* A study undertaken in both Canada and the United States was counted twice. ^±^ General Health referred to Public Health professionals, Psychologists, health researchers, and otherwise unspecified health professionals or practitioners. ^¥^ Allied Health Professionals referred to Dentists, Pharmacists, Occupational Therapists, Physiotherapists, Audiologists and Speech Language Therapists, and Radiologists. ^α^ Education refers both to those in the field of Education and to university students/staff in unspecified fields.

## Appendix D

**Table A4:** Citations for Indigenous Cultural Safety Training Interventions.

Indigenous Cultural Safety Training Interventions (*n* = 69)	
Indigenous People(s) Involved in Development Process	[40,42,45,47,49,50,51,54,56,57,65,66,68,70,73,76,78,83,84,85,86,87,89,91,93,94,100,101,103,109,110,111,112,114,115,116,118,121,122,125,131,144,147,150,151,153,155,160]
Training Modalities	
Teaching/lectures	[40,42,47,49,51,54,57,65,83,84,86,91,92,94,101,103,109,121,129,132,144]
Workshop	[55,65,68,70,77,80,85,87,88,101,110,111,118,125,141,144,147,155]
Discussion	[40,47,49,50,55,80,84,85,93,109,110,118,119,129,142,147]
Immersive experience/ community visit	[40,49,51,65,84,86,100,110,114,116,147,153,160]
Storytelling/yarning	[66,74,80,88,93,101,109,121]
Reflective exercise/practice	[85,93,97,101,109,110,118,129]
Online content	[74,83,91,93,112,127,129,167]
Video/videocast	[40,54,76,83,85,97,101,115]
Clinical placement	[51,78,122,147,150,151,160]
Case-study	[25,51,54,55,76,118,129]
Tutorial	[47,54,97,103,112,144,147]
Workbook/readings	[40,42,93,110,125,142,153]
Elder/mentor/community support	[56,84,86,93,125,153]
Course-based modules	[56,84,86,93,125,153]
Group work	[25,50,65,84]
Drama/role-playing exercise	[149,155]
Visit to gallery/museum	[40,109]
Post-intervention support Provided	[40,42,56,67,68,131,141,160]
Training Timeline	
1–5 days/sessions	[25,45,50,65,68,70,85,94,111,113,116,118,141,149,155]
Partial course (8–25 h), 1 semester	[49,54,57,80,82,83,88,92,97,99,112,121]
1 Lecture/workshop/cultural visit	[55,77,87,89,101,109,125,132,141,155]
1–5 week(s) of immersion	[100,110,114,122,151,160,167]
4 years of embedded content, bachelor’s degree	[50,51,129,131,147,153]
6–10 days/sessions	[59,65,85,91,103,122]
3–12 months of immersion	[97,150]
Full course (42 h), 1 semester	[47,144]
Partial course (50–52 h), 2 semesters	[40,78]
Intensive course content (148 h), 2 semesters	[42]
2 years of content, embedded in health service organization	[119]
Not mentioned	[43,44,56,73,74,76,84,93,115,131,134,142,161]
Who Delivered the Training	
Professors/administrators, university	[25,40,42,51,54,65,73,74,76,80,82,85,93,94,101,112,115,116,118,121,122,125,129,160,167]
Clinicians/clinical instructors, health	[40,45,49,51,56,65,68,70,80,85,87,98,110,114,116,121,131,132,150,167]
Indigenous elder/ mentor/cultural educator/ community/ community organization	[56,86,94,100,101,103,110,111,112,114,116,118,121,122,125,150,151,153,160]
Lecturers/educators, university/college	[47,55,57,83,84,86,89,91,93,97,112,116,141,144,149]
Researchers	[55,70,86,101,118]
Allied health professional	[66,147,155]
Consultants	[45,68,119]
Not mentioned	[50,124]
Non-clinician staff, health	[25]
Indigenous people(s) involved in delivering intervention ^	[45,47,54,55,56,57,65,66,68,70,80,85,86,87,89,93,94,97,100,103,109,111,112,114,115,116,118,121,125,144,150,151,153,155,160]

^ Indigenous peoples involved in delivering cultural safety training include both those categorized as “Indigenous Elder/Mentor/Cultural Educator/Community/Community organization” as well as those in other relevant categories listed immediately above.

## Appendix E

**Table A5:** Citations for Indigenous Cultural Safety Training Intervention Evaluations.

Details of Indigenous Cultural Safety Training Intervention Evaluations (*n* = 61)	
Evaluation Focus/Objectives	
General learning experiences, perceptions, needs, and/or preferences	[40,43,49,59,66,70,78,80,109,110,111,112,115,116,122,131,141,144,151]
Indigenous community members’ perceptions of intervention	[40]
Intervention Outcomes	
Learning experiences, outcomes, and/or barriers	[43,49,57,84,85,99,100,101,103,113,114,115,116,119,131,155]
New skills/behaviours/practices	[59,65,89,94,107,114,115,119,121,123,125,132,140,151,155]
New knowledge/awareness/attitudes/confidence	[55,88,89,92,99,100,113,118,119,155,160]
Perceptions of/attitudes toward Indigenous peoples/their health issues	[99]
Learners’ receptivity/resistance to intervention content	[97]
Intervention and/or Behaviour Change Processes	
Intervention planning and implementation processes	[42,74]
Behaviour change processes	[121,132]
Relationship Between Exposure and Outcomes	
Between learners’ perceptions of Indigenous peoples and their intervention participation	[47]
Intervention context	
Enabling factors within organizational context	[113,118,123,125,140]
Impact on organization processes and priorities	[59,113,119]
New organizational policies, positions, capacity, and mandate	[103,107]
Methodological approaches	
Qualitative and quantitative methods	[40,43,54,55,59,65,68,70,74,78,80,82,84,85,89,94,97,99,101,107,110,114,115,116,121,125,131,141,144,151,155,160]
Qualitative methods only	[42,49,51,57,62,64,66,72,73,86,88,100,103,106,109,112,119,132,140,161]
Quantitative methods only	[47,69,92,113,118,123,153,163,165]
Research methods	
Pre-Intervention	
Pre-survey	[74,78,80,89,94,97,99,101,115,119,123,131,160]
Pre-survey, with open-ended questions	[55,85,107,113,125,155]
Pre-focus group	[131,161]
Pre-test	[118]
2. Mid-Intervention	
Mid-researcher observations/reflections	[80,97,100,119]
3. Post-Intervention	
Post-survey	[40,43,47,55,57,74,78,80,84,92,94,97,110,111,115,116,119,122,123,131,141,144,151,160]
Post-surveys, with open-ended questions	[57,59,65,66,70,89,99,101,103,112,116,153]
Participant Interview	[19,40,43,49,88,97,100,115,119,122,140,144]
Oral/written learner feedback	[78,84,85,94,109,116]
Post-focus group	[40,94,100,110,131,161]
Learners’ reflections/case scenarios	[100,116,132,160]
Learner journal entries/digital storytelling	[74,80,110]
Post-test	[118]
Talking circles	[116]
Analysis of developed curriculum	[42]
Post-researcher observations/reflections	[115]
4. Delayed Post-Intervention	
Delayed post-survey (3–55 months)	[100,121]
Delayed learners’ reflections (2–3 weeks)	[100]
Limitations stated for chosen methods	[42,47,54,55,57,59,62,65,73,74,80,82,85,88,89,92,99,101,103,106,107,114,115,118,119,123,125,131,140,141,151,155,163]
